# Prevalence of Pseudoexfoliation Syndrome and Pseudoexfoliation Glaucoma in Upper Egypt

**DOI:** 10.1186/1471-2415-11-18

**Published:** 2011-06-27

**Authors:** Tarek A Shazly, Abdelsattar N Farrag, Asmaa Kamel, Ashraf K Al-Hussaini

**Affiliations:** 1 Department of Ophthalmology, Assiut University Hospital, One Assiut University Campus, Assiut, 71526 Egypt; 2 Department of Ophthalmology, Massachusetts Eye and Ear Infirmary/Harvard Medical School, 243 Charles St, Boston, MA, 02114 USA; 3 Department of Ophthalmology, Al-Hussaini Hospital, 23th July St. Assiut, 71111 Egypt

## Abstract

**Background:**

Pseudoexfoliation (PXF) is a recognized risk factor for developing cataract, glaucoma and lens dislocation. PXF is also associated with increased risk of complications during cataract surgery due to poor mydriasis and zonular weakness. The aim of this study is to report the prevalence of pseudoexfoliation among Upper Egyptians attending the ophthalmology clinic of Assiut University Hospital.

**Methodology:**

A retrospective, chart review study conducted in the period from February 2002 to August 2009. A total of 7738 patients aged 40 years or older attending the general ophthalmic clinics were included in this study. A detailed evaluation including ophthalmic and general history, slit lamp biomicroscopy, intraocular pressure measurement, gonioscopy and dilated eye examination were performed. Patients with pseudoexfoliative material on the anterior lens surface and ⁄ or the pupillary margin in either or both eyes were labeled as having PXF.

**Results:**

Out of the 7738 patients included, three hundred twenty (4.14%) subjects had PXF. Mean age of PXF group was 68.15 years (SD 8.16, range 40-92 years). PXF was bilateral in 82.2% of cases. It was significantly associated with cataract, glaucoma and hearing loss. Of the PXF patients, 65% had cataract, 30.3% had glaucoma and 8.1% had hearing loss.

**Conclusion:**

Pseudoexfoliation appears to be a common disorder in older individuals in Upper Egypt.

## Background

Pseudoexfoliation syndrome (PXF) was first reported by Lindberg in 1917 in a Finnish population [1]. It is characterized by the deposition of a distinctive fibrillar material in the anterior segment of the eye. Pseudoexfoliation syndrome is frequently associated with open angle glaucoma, known as pseudoexfoliation glaucoma, which is the most common identifiable form of secondary open angle glaucoma worldwide [2]. Pseudoexfoliation is a known risk factor for developing cataracts [3]. Complicating factors such as poor mydriasis, zonular weakness, corneal endothelial dysfunction, higher rate of vitreous loss, capsular phimosis, and opacification have all been reported after cataract surgery [4,5]. Pseudoexfoliation is considered to be a systemic disorder, pseudoexfoliative material has been reported in lungs, skin, liver, heart, kidney, gallbladder, blood vessels, extra ocular muscles and meninges [6]. An association between PXF and sensorineural deafness has been reported [7-11].

Pseudoexfoliation is rarely seen before the age of 40, and its prevalence increases markedly with age [12]. Although it occurs in virtually every area of the world, a considerable racial variation exists. Framingham study showed that the prevalence of PXF was 1.8% [13]. In another study of subjects over 60 years in various ethnicities, prevalence rates ranging from 0% in Greenland Eskimos to 21% in Icelanders were noted [14]. In northern/western European countries including England, Germany, and Norway prevalence of 4.0%, 4.7%, and 6.3% have been reported respectively [15].

Epidemiological studies of PXF have been done in some areas in the Middle East, namely in Jordan and Yemen [16,17], but there is no data available on prevalence of PXF in Egypt.

Egypt is one of the most populous countries in Africa and the Middle East, with a population approaching 80 million. Assiut University Hospital (AUH) is a tertiary medical centre in Assiut - the most populous governorate of Upper Egypt -which serves a large number of Egyptians from all over Upper Egypt. The aim of this hospital-based study is to estimate the prevalence of PXF, provide a descriptive analysis and to assess whether PXF is associated with cataract, glaucoma, hearing loss, systemic arterial hypertension and/or diabetes mellitus.

## Methods

The charts of 7738 consecutive patients aged 40 years or older who attended the general ophthalmic clinics of Assiut University Hospital in the period from February 2002 to August 2009 were included in this retrospective study.

The study protocol was approved by the medical ethics committee of Assiut University.

Relevant details in medical and ocular history were obtained from each patient including; history of systemic arterial hypertension, diabetes mellitus, hearing loss, intraocular surgery, visual problems, amblyopia and use of corrective glasses and/or contact lenses. All patients underwent complete ocular examination conducted by a senior experienced ophthalmologist (AKA) included slit lamp biomicroscopy, intraocular pressure measurement, gonioscopy, and dilated fundus examination.

Pseudoexfoliation was diagnosed clinically by the presence of typical pseudoexfoliation material (PXM) at the pupil border on undilated examination, on anterior lens capsule on dilated examination, or on the trabecular meshwork on gonioscopy, with or without Sampaolesi's line and pigment deposition in angle and/or corneal endothelium.

Cataracts were graded at the slit lamp with respect to nuclear opalescence (NO: 0.1-6.9), nuclear color (NC: 0.1-6.9), cortical (C: 0.1-5.9) and posterior subcapsular (P: 0.1-5.9) cataract, using the Lens Opacities Classification System (LOCS) III [18]. According to the average LOCS III value (average of NO, NC, C, P), lens opacities were defined as no cataract (avLOCS<1.5), and cataract (avLOCS≥1.5).

Diagnosis of glaucoma was made if: intra ocular pressure (IOP) ≥ 22 mm Hg in either eye; vertical cup disc ratio (VCDR) ≥ 0.7 or a cup disc ratio (CDR) asymmetry ≥ 0.2; and focal thinning, notching, or a splinter hemorrhage. Glaucoma was defined based on the International Society of Geographical and Epidemiologic Ophthalmology (ISGEO) classification [19].

Detailed history of diabetes mellitus, systemic arterial hypertension and hearing loss was specifically obtained in PXF and non-PXF subjects.

The statistical analysis was performed using the Statistical Program for the Social Sciences Version 16.0 (SPSS, Inc, Chicago, IL, USA).

Means, standard deviations (SDs) and 95% confidence intervals (CIs) were obtained. A *p*-value of < 0.05, measured by Pearson's chi-square test, was considered to indicate statistical significance.

Multiple logistic regression models were used to examine the association between PXF with cataract and glaucoma, while adjusting for gender and age.

Patients were divided into two groups; PXF and non-PXF. For each group the sex distribution, mean age and standard deviation (SD) were calculated. The total number of eyes studied was 7738. The PXF group included 320 eyes and the non-PXF group included 7418 eyes. Frequencies of cataract, glaucoma and hearing loss in both groups were estimated.

## Results

Out of 7738 patients enrolled, 320 were diagnosed with PXF. Thus the prevalence of PXF in those ≥40 years was 4.14%. The age-adjusted prevalence of PXF in those ≥60 years was 6.02 (95% CI: 4.70-7.51) in those ≥60 years of age. We calculated the age-specific standardized rates of PXF by using the U.S. population as a standard.

Of the 320 PXF patient included, 132 (41.25%) were female and 188 (58.75%) were male. The association between PXF and gender was not statistically significant (*p *= 0.09).

Mean age in the PXF group was 68.15 years (SD 8.16, range 40-92 years). Prevalence of PXF increased with age and was highest among subjects aged > 80 years. Approximately 90% of all PXF patients were over 60 years old (table 1).

**Table 1:** Age and gender distribution of the study group

Age group	No of males studied	Number of male PXF patients (percentage)	No of females studied	Number of female PXF patients (percentage)	*p*-value*
**40-49**	43	3 (7%)	24	1 (4.1%)	>0.05

**50-59**	532	13 (2.4%)	360	14 (3.9%)	>0.05

**60-69**	1553	80 (5.2%)	1288	45 (3.5%)	>0.05

**70-79**	1633	70 (4.3%)	1427	59 (4.1%)	>0.05

**80-89**	484	20 (4.1%)	266	11 (4.1%)	>0.05

**90-99**	70	2 (2.9%)	59	2 (3.4%)	>0.05

***Total***	**4315**	**188 (4.35%)**	**3424**	**132 (3.9%)**	**>0.05**

* Chi Square test.

**Figure 1:**
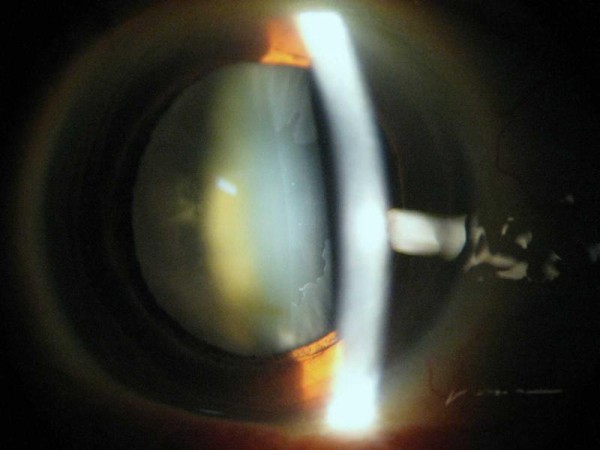
**An eye with pseudoexfoliation showing the dandruff like pseudoexfoliation material on the surface of the cataractous lens capsule**.

Unilateral PXF was noted in 17.8% of the PXF group and bilateral PXF was noted in 82.2% (figure 1).

**Figure 2:**
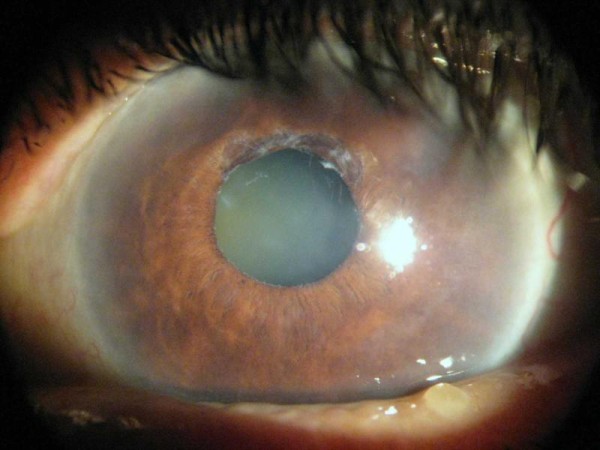
**An eye with pseudoexfoliation showing the dandruff like pseudoexfoliation material on the pupillary edge**.

Cataract was found in 65% of eyes with PXF (figure 2), but in only 42.5% of non-PXF eyes (*p *< 0.001), indicating a strong association between cataract and PXF (Table 2). During the study period, 3289 patients underwent cataract surgery in one or both eyes, of which 6.32% had PXF.

**Table 2:** Logistic regression model analysis of the association between PXF with cataract, glaucoma and hearing loss while adjusting for gender and age.*

	PXF, n (%)	Non-PXF, n (%)	PXF:Non-PXF Odds ratio	*p*-value
**Cataract**			1 : 15.1	

Present	208 (65)	3149 (42.5)		

Absent	112 (35)	4269 (57.5)		

Total	320 (100)	7418 (100)		< 0.001

**Glaucoma**			1 : 2.52	

Present	97 (30.31)	244 (3.3)		

Absent	223 (79.7)	7174 (96.7)		

Total	320 (100)	7418 (100)		< 0.001

**Hearing loss**			1 : 6.6	

Present	26 (8.1)	171 (2.3)		

Absent	294 (91.9)	7247 (97.7)		

Total	320 (100)	7418 (100)		< 0.001

PXF = pseudoexfoliation.

*Age adjustment was performed using the population estimates for the U.S. for the year 2000 as the standard.

Glaucoma was found in 30.31% of eyes in the PXF group, but in only 3.3% of eyes in the non-PXF group. The association between PXF and glaucoma was statistically significant (*p *< 0.001), as shown in Table 2.

Out of the 320 patients, 34 (10.6%) were already diagnosed with psedoexfoliation glaucoma and 63 patients (19.7%) had IOP of more than 21 in one or both eyes and have not been evaluated for glaucoma before.

Hearing loss was documented in 8.1% of 320 PXF patients and in only 2.3% of 7418 non-PXF. This association between PXF and hearing loss was statistically significant (table 2).

Significant corneal opacities were present in 7.9% of the PXF patients and 6.1% of non-PXF patients. The difference between the 2 groups was not statistically significant (*p *> 0.05).

We found no statistically significant association between PXF and diabetes mellitus (*p *= 0.18) or systemic arterial hypertension *p *= 0.25.

## Discussion

The reported prevalence rate of PXF syndrome in different populations shows extensive variations. Prevalence rates of as low as 0% in Eskimos [20], and as high as 38% in Navajo Indians [21] were reported.

In a hospital based study conducted in Jordan, the prevalence of pseudoexfoliation among patients aged 40-90 years was 9.1% [17]. In another study from Yemen the point prevalence of PXF among patients undergoing cataract surgery was 19.53% [16].

To the best of the authors' knowledge, there are no previous reports on the prevalence or characteristics of PXF in Egypt. The prevalence of pseudoexfoliation among individuals aged 40 years or older in this study is 4.14%.

One of the limitations of this study is being a hospital-based rather than a population-based study. Over or under-estimation of the prevalence of PXF and or co-morbidities associated with PXF may be attributed to the hospital based nature of the study.

In agreement with findings in other reports, our study showed an increase in the prevalence of PXF with advancing age [22-24].

PXF was more common among males (188) than in females (132). A similar finding was reported in the prevalence of PXF among patients undergoing cataract Surgery in Yemen [16]. We found that PXF was bilateral in the majority of cases (82.2%), comparable to what Tiliksew et al. found [24].

A significant association between PXF and cataract was also found in our study and is compatible with findings in other studies [23,24].

The age-adjusted PXF rate (direct standardization using the population estimates for the U.S. for the year 2000 as the standard) in our study population for those 40 years of age or older was similar to the age-standardized rates of PXF reported in south India (3.01%) reported by Thomas et al. [25], in the Chennai study (4.9%) [23], and Blue Mountains Eye Study (2.3%), [26] and higher than the rates in the Framingham Eye Study (1.9%), [27] and Visual Impairment Study (0.98%) [28]. However, the age-specific standardized PXF rates in other population-based studies--one from southern India [29], central Iran [30] and from Crete (Greece) [31,32]--were high (7.6%, 9.4% and 16.1% respectively) in comparison to those in our study.

A strong relationship between glaucoma and PXF is known [33]. Subjects with PXF had a two- to threefold increased risk for glaucoma according to the Blue Mountains Eye Study [26]. Other studies have demonstrated that eyes with PXF had higher mean IOP than eyes without PXF [13,26]. Moreover, Topouzis F et al. reported increased likelihood of glaucoma at the same IOP in subjects with PXF [34]. Our study is consistent with the above as we found an increased risk for glaucoma in patients with PXF. Our finding that 30.31% of eyes with PXF had glaucoma reflects a comparable proportion to that reported by Al-Bdour et al. in Jordan [17], yet higher that that found by the Blue Mountains Eye Study, which found incidences of 14.2% [26]. This finding may reflect an overestimation, which is one of the limitations of hospital-based studies.

One of the limitations of our study is that hearing loss was assessed via detailed history taking. Some of the patients had audiometry documented sensorineural hearing loss, while others didn't undergo audiometric studies.

In our study hearing loss was found in 8.1% of PXF patients. This association between PXF and hearing loss was statistically significant (table 2). Cahill *et al. *reported that a large proportion of patients with PXF have sensorineural hearing loss in comparison to age-matched controls, regardless of whether or not there is associated glaucoma [7]. This was confirmed by other studies from different Saudi Arabia, Canada, Turkey and Iran [8-11]. Furthermore, in a recent study Turgut et al. reported high prevalence of asymptomatic vestibular dysfunction among patients with PXF [35].

Although the material reported here has many limitations, it adds some new information on the prevalence and characteristics of PXF in a region where data on PXF are scarce.

## Conclusion

In conclusion we found the prevalence of PXF among Upper Egyptian individuals aged 40 years or older to be 4.14%. This rate is similar to other studies conducted in south India, the Chennai study, and Blue Mountains Eye Study. Ophthalmologists in Egypt should focus on the detection of PXF, especially considering the risks for operative complications related to PXF and the higher prevalence of glaucoma among PXF patients.

## Competing interests

The authors declare that they have no competing interests.

## Authors' contributions

TAS participated in study design, performed the statistical analyses and drafted the manuscript. ANF and AK participated in study design and in the data collection. AKA examined the patients participated in study design and critically appraised the manuscript. All authors read and approved the final manuscript.

## Pre-publication history

The pre-publication history for this paper can be accessed here:

http://www.biomedcentral.com/1471-2415/11/18/prepub
